# A prion-like mechanism for the propagated misfolding of SOD1 from in silico modeling of solvated near-native conformers

**DOI:** 10.1371/journal.pone.0177284

**Published:** 2017-05-04

**Authors:** Eamonn F. Healy

**Affiliations:** Department of Chemistry, St. Edward’s University, Austin, Texas, United States of America; Russian Academy of Medical Sciences, RUSSIAN FEDERATION

## Abstract

A prion-like mechanism has been developed to explain the observed promotion of amyloid aggregation caused by conversion of structurally intact SOD1 to a misfolded form. Superoxide dismutase [Cu-Zn], or SOD1, is a homo-dimeric protein that functions as an antioxidant by scavenging for superoxide. The misfolding and aggregation of SOD1 is linked to inherited, or familial, amyotrophic lateral sclerosis (FALS), a progressive and fatal neurodegenerative disease. Aberrant SOD1 folding has also been strongly implicated in disease causation for sporadic ALS, or SALS, which accounts for ~90% of ALS cases. Studies have found that mutant, misfolded SOD1 can convert wtSOD1 in a prion-like fashion, and that misfolded wtSOD1 can be propagated by release and uptake of protein aggregates. Here it is demonstrated that enervating the SOD1 electrostatic loop can lead to an experimentally observed gain of interaction (GOI) responsible for the formation of SOD1 amyloid-like filaments. This enervation is caused in turn by the formation of transient, non-obligate oligomers between pathogenic SOD1 mutants and wt SOD1.

## Introduction

Amyotrophic lateral sclerosis (ALS) is a progressive and fatal neurodegenerative disease. Aberrant superoxide dismutase (SOD1) oligomerization has been strongly implicated in disease causation, even for sporadic ALS, or SALS, which accounts for ~90% of ALS cases. While it is known that oligomeric assemblies of both wild type (wt) and mutant SOD1 are precursors to the larger and detergent-insoluble aggregates, the structural events that trigger oligomerization remain elusive. In addition to being classified as a proteinopathy, studies have found that mutant and misfolded SOD1 can convert wtSOD1 in a prion-like fashion [1], and that misfolded wtSOD1 can be propagated by release and uptake of protein aggregates [2]. Thus a case has been made that ALS exhibits the characteristics associated with the prion paradigm [3], and that mutant or misfolded SOD1 can function as an infectious protein.

**Fig 1:**
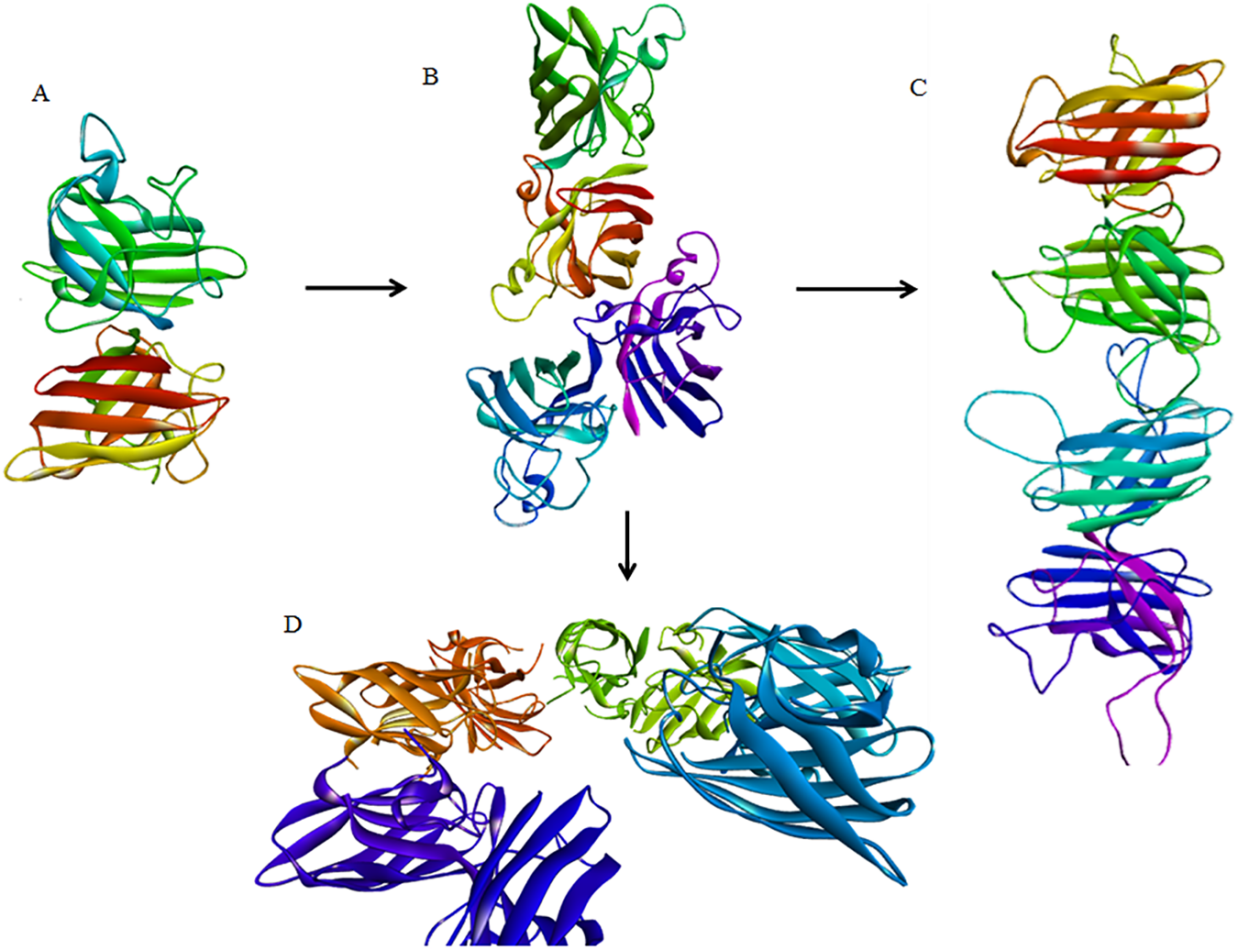
**A** The wt SOD1 dimer; **B** The non-obligate SOD1 tetramer predicted by protein-protein docking; **C** The apo H46R SOD1 mutant linear filament as characterized in reference 21; **D** The helical filament for the Zn-H46R SOD1 mutant as characterized in reference 21.

By focusing on solvent-exposed intramolecular backbone hydrogen bonds as physico-chemical descriptors for protein packing, we have previously developed a role for transient, non-obligate oligomers in the formation of aberrant protein aggregates [4]. Solvent-exposed intramolecular backbone hydrogen bonds, or dehydrons, are vulnerabilities, or structural defects, in the packing of a wide array of proteins [5,6]. Exposure of such dehydrons to an aqueous environment weakens protein secondary structures [7,8], and in turn excluding solvent from protein regions containing exposed hydrogen bonds can be a determinant factor in protein-protein interactions (PPI) [9], and protein subunit assembly [10]. For wtSOD1 and a range of mutants there are three relatively well defined regions of underwrapped, solvent-exposed backbone hydrogen bonds. One of these regions lies at the subunit interface and is thus dehydrated and stabilized through formation of the dimer. The other two areas correspond to the electrostatic loop, and the DKDG loop connecting the 5^th^ and 6^th^ β-sheets. The SOD1 tetramer predicted by protein-protein docking, and shown in Fig 1B, serves to protect, or desolvate, these areas of vulnerability that are shared by wtSOD1 and a variety of pathogenic mutants.

A well-defined association has been established between subunit flexibility and the assembly of protein complexes [11], and more recently it has been observed that a destabilizing mutation in the enzyme dihydrofolate reductase (DHFR) leads to a functional tetramerization of the otherwise monomeric enzyme [12]. The dramatic subunit asymmetry reported for the G37R mutant crystal structure, as well as higher crystallographic B-factors measured for the electrostatic loop residues [13], identify it as markedly less rigid than wtSOD1. For the tetrameric complex characterized as the ASU for the G85R SOD1 mutant (pdb entry 2ZKX) residues 126–139 of chain D, the subunit distal to the dimer-dimer interface, are missing, also indicating significant disorder. Computational studies have indicated that SOD1 mutants have a significantly altered pattern of mobility compared to wtSOD1 [14], with all mutants studied found to be more flexible than the wild type, and with the highest flexibility computed for those residues within the electrostatic loop. Thus the PPIs that would accompany oligomerization could serve to compensate for the destabilizing effects of SOD1 mutations. If such oligomers involve both wt and mutant SOD1 components, expected due to near identical topologies and dehydron distributions, then these PPIs could also provide a mechanism for mutant SOD1 to induce misfolding in the wt homodimer.

The conformational change hypothesis [15] is based on the concept that partial protein unfolding is required to initiate the aggregation of a globular protein. These conformational states, while being thermodynamically distinct from the native state, can be structurally similar to it and accessed by thermal fluctuations [16]. We have used landscape energy theory to demonstrate how the dimer-dimer interaction observed in the assymetric unit cell (ASU) of the G85R SOD1 crystal structure might result in two conformational-change scenarios, one local to the electrostatic loop at the complex interface, and a second displacement at the electrostatic loop of the other dimeric subunit [17]. The energy landscape theory of protein folding is a statistical model of a protein as a minimally frustrated heteropolymer with a rugged funnel-like landscape biased toward the native state [18]. The principle of minimal frustration still allows that some energetic frustration may be present in a folded protein, with this local violation facilitating motion of the protein around its native basin. As such the residual frustration may be fundamental to conformational change and protein function [19,20]. Thus a non-obligate tetramer formed from mutant and wt SOD1 homodimers, and predicted by protein-protein docking, could initiate wtSOD1 misfolding by inducing localized conformational change in those domains proximate to the oligomeric interface.

**Fig 2:**
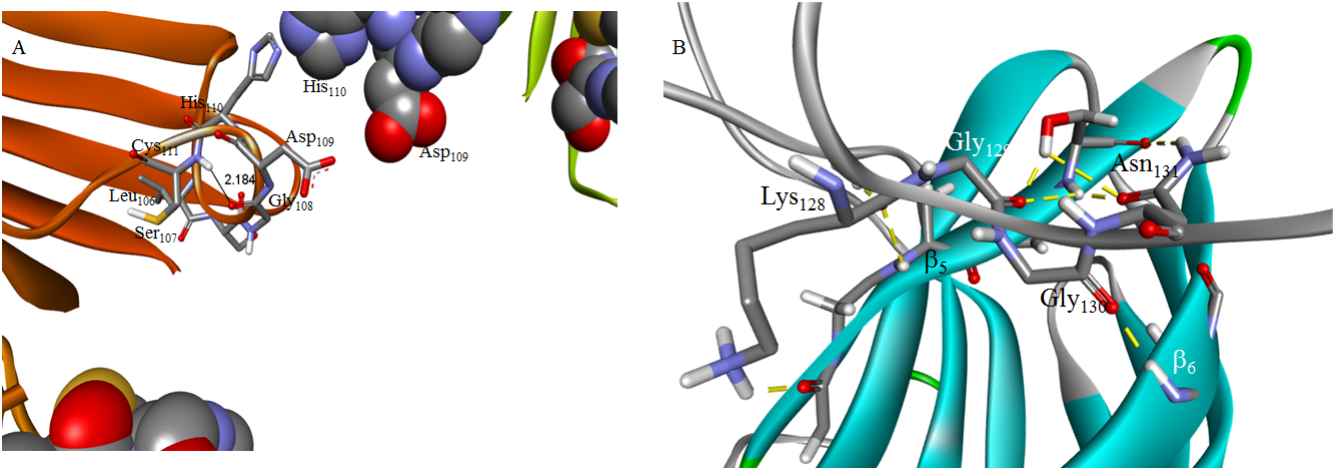
**A** the Zn-H46R helical filament cavity with the solvent-accessible hydrogen bond between the Cys_111_ donor amide and the Gly_108_ acceptor carbonyl shown as stick figure, and the shielding LSGDHC sequence in the β_6_-β_7_ loop of an adjacent dimer, shown as spacefilled atoms; **B** The hydrogen-bonding contacts at the electrostatic loop/β_5_-β_6_ cleft interface of the apo H46R SOD1 mutant linear filament refined as described in reference 22.

An apo H46R SOD1 crystal structure has been obtained consisting of amyloid-like filaments in which adjacent dimers interact through a gain-of-interaction (GOI) contact between the unstructured electrostatic loop of one dimer and the cleft defined by edges of the β_5_ and β_6_ strands of an adjacent dimer [21], Fig 1C. A second crystal structure was also characterized for H46R, this time for the mutant containing Zn but not copper, that reflects a GOI between the unfolded zinc-binding loop in one dimer and the β_5_-β_6_ cleft of an adjacent dimer. In contrast to the linear filament seen in the apo mutant, this GOI facilitates a helical filament as shown in Fig 1D. Repetition of this GOI results in the formation of a hollow tube with an inner water filled cavity having a diameter of about 30 Å. As was noted in the original characterization of the apo H46R and Zn-H46R filaments such amyloid pores have also been observed in pathogenic mutants for both Alzheimer’s and Parkinson’s disease. We have previously used homology modeling and molecular dynamics (MD) to supply the missing structural elements of the linear amyloid filament [22]. The completed filament, showing details of the GOI between the electrostatic loop of one dimer and the cleft defined by edges of the β_5_ and β_6_ strands of the adjacent dimer, is shown in the Fig 2B. Recently the β_6_-β_7_ loop residues _106_LSGDHC_111_ have been identified as an aggregation-initiation sequence for SOD1 [23]. From Fig 2A it can be seen that in the inner wall of the helical amyloid filament of Zn-H46R the _106_LSGDHC_111_ residues in the β_6_-β_7_ loop of one dimer are proximate to the _106_LSGDHC_111_ residues of the adjacent dimer. This alignment could help explain the inhibition of reduced and demetallated G93A SOD1 fibril elongation by peptides containing the LSGDHC sequence. These results highlight well-characterized GOIs involving locally unfolded SOD1 electrostatic and zinc-binding loops that facilitate the formation of SOD1 amyloid filaments with structures consistent with recent experimental findings.

Correlated motion among domains within, and between, subunits of SOD1 has been previously highlighted as a possible mechanism for dissipating thermal or environmental energy perturbations [14]. Enervating the electrostatic loop through the dimer-dimer contact at the interface in Fig 1B would prevent such energetic dissipation leading to localized thermal fluctuations, thus promoting local unfolding. The analysis presented here uses MD simulations and landscape energy theory to demonstrate how such enervation can generate non-native SOD1 conformers by promoting local unfolding of both the electrostatic and zinc-binding loops. Quantum mechanical characterization of a low energy conformer demonstrates how changing solvent accessibility within both the electrostatic and zinc-binding loops can lead to the introduction of unfavorable electrostatic contacts between key residues. How these localized changes could facilitate the GOI contacts characterized in the H46R filaments is discussed

## Materials and methods

The crystal structures for wild type (wt) and G93A mutant of human superoxide dismutase are available from the RCSB (www.rcsb.org) as PDB entries 2C9V [24] and 2WZ6 [25]. After adding hydrogens all proteins were subjected to a short energy minimization using the CHARMm force field [26]. Protein alignments and superimposition were done using the MODELER protocol as implemented in the Discovery Studio program suite.

For the Molecular dynamic (MD) simulations SOD1 dimers were immersed in an orthorhombic cell of TIP3P explicit water molecules and neutralized with counterions, and the system was minimized by steepest descent and conjugate gradient. The complex was heated to 300 K over 100 ps, and equilibrated at 300K for 500 ps. A 2 ns production was run at 300K with, and without, the application of a harmonic restraint to the residues of the electrostatic loop of one subunit (residues 121–142). The SHAKE algorithm was employed to keep bonds involving hydrogen atoms at their equilibrium length, allowing the use of a 2 fs time step. Complete MD Protocol is available at http://dx.doi.org/10.17504/protocols.io.hqnb5ve.

Frustration contacts were calculated using the “Frustratometer Server” hosted by the Universidad de Buenos Aires, and available for use at http://www.frustratometer.tk/. This calculation of localized frustrated interactions within a protein involves the systematic perturbation of the protein sequence, and the computation of the resulting energy change using the Associative Memory Hamiltonian water-mediated potential (AMW) energy function [20]. A histogram of the energy of decoys is compared to the native energy. A contact is defined as “minimally frustrated” if its native energy is at the lower end of the distribution of decoy energies. Specifically these are contacts having a measured Z score, for the energy of the decoys compared to the energy of the native pair, of 0.78 or higher, signifying that the majority of other amino acid pairs in that position would be unfavorable by more than one standard deviation of that distribution. Conversely, a contact is defined as “highly frustrated” if the energy is at the other end of the distribution with a Z score of lower than -1, signifying that most other amino acid pairs at that location would be more favorable for folding than the native ones by more than one standard deviation of that distribution. If the native energy is in between these limits the contact is defined as “neutral”.

Solvent-exposed intramolecular backbone hydrogen bonds, or dehydrons, are identified as backbone hydrogen bonds “wrapped” by a number of non-bonded, carbonaceous groups, ρ, contained within a domain centered on the interacting residues, that is at or below <ρ> minus one root mean squared deviation. Dehydron analysis of the G93A mutant identifies the hydrogen bond between the Cys_111_ donor amide and the Gly_108_ acceptor carbonyl as extremely vulnerable with a ρ = 10 compared to a mean value of ρ of 24 for all hydrogen bonds in pdb entry 2WZ6.

QM/MM calculations were carried out at the DFT (B3LYP//DNP)/CHARMm and (B3LYP//TNP)/CHARMm levels, using the Becke, three-parameter, Lee-Yang-Parr (B3LYP) exchange-correlation functional, at both the double numerical polarized (DNP) the triple numerical polarized (TNP) basis set levels, and the CHARMm force field. The QM region was defined as the His_71_ and Asp_124_ residues together with the bound water molecule. In calculating the free energy of solvation a TΔS value of 7.5 kcal mol^-1^ per bound water molecule was used as an entropic correction. This value represents the average entropic contribution per water molecule calculated at 298K for a variety of water clusters using complete basis set model chemistries [27].

## Results and discussion

**Fig 3:**
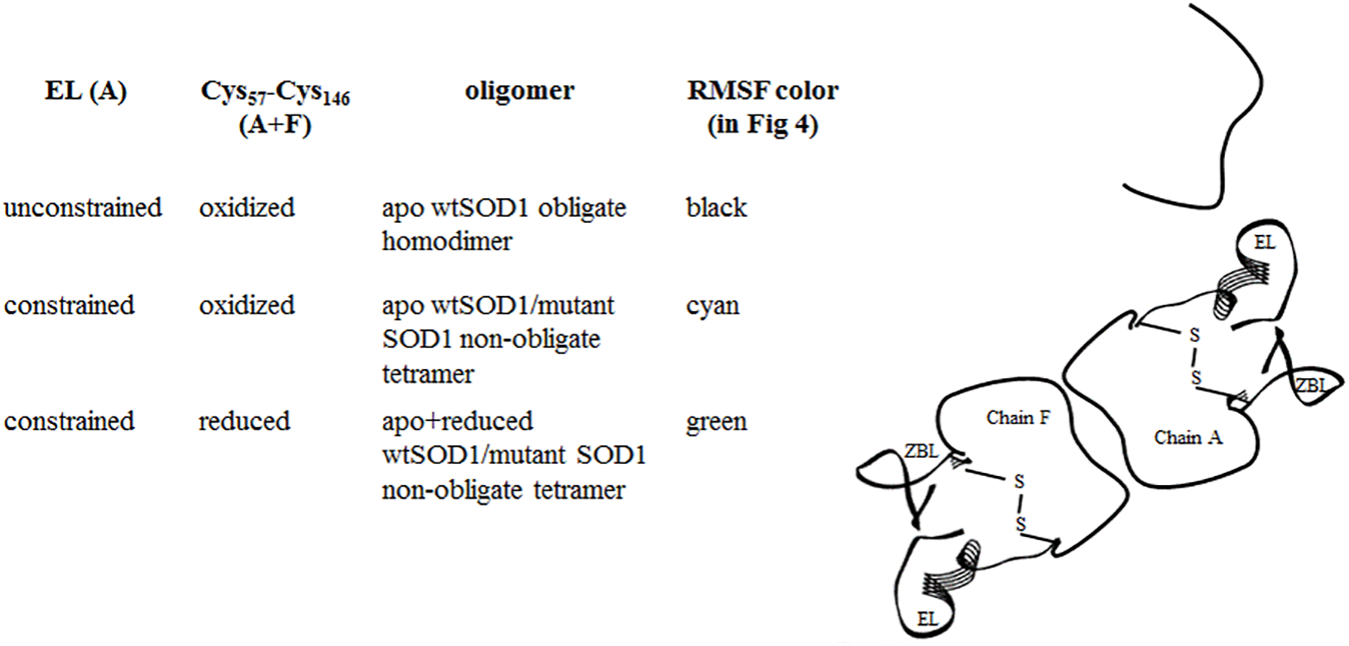
Schematic showing how MD simulations with the application of constraints are used to simulate the structural consequences of SOD1 oligomerization. Harmonic constraints applied to the electrostatic loop (EL) of subunit A of the SOD1 homodimer simulates the structural consequences of oligomerization by enervating the key domain at the interface. Oxidizing and reducing conditions simulated by maintaining or eliminating the Cys_57_-Cys_146_ disulfide bridge in both subunits (A and F), with the wtSOD1 obligate dimer simulated by an MD run without any constraints. Each simulated scenario is associated with the appropriate color for the root mean square fluctuations (RMSF) shown in Fig 4.

The schematic in Fig 3 identifies how MD simulations, coupled with the application of constraints, can be used to simulate the structural consequences of SOD1 oligomerization. Our prior frustration analysis of the G85R SOD1 mutant [17] demonstrated how the oligomer characterized in the ASU enervated the electrostatic loop at the dimer-dimer interface through elimination of the intra-loop highly frustrated contacts that correlate with loop mobility. Harmonic constraints applied to the electrostatic loop (residues 121–142) of one subunit of the SOD1 homodimer therefore simulate the structural consequences of oligomerization by enervating the key domain at the interface. This effect can be modelled under both oxidizing and reducing conditions by maintaining or eliminating a key disulfide bridge associated with subunit integrity. The mobility of the residues in the wtSOD1 obligate dimer, obtained from a simulation without any constraints, serves as a reference.

**Fig 4:**
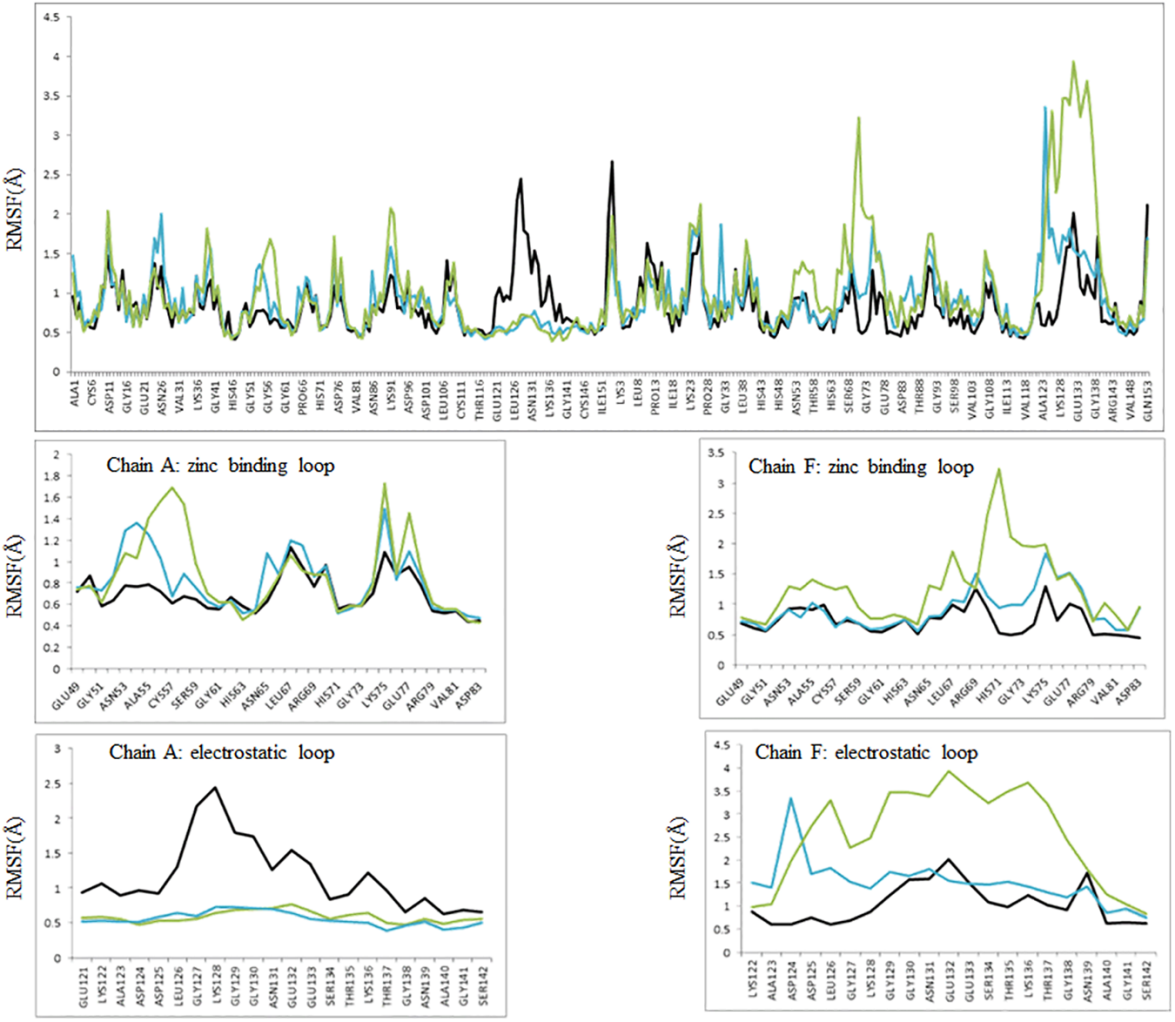
Root mean square fluctuations (RMSF) for the 2 ns production runs of apo wtSOD1dimer (black), apo wtSOD1 with the application of a harmonic restraint to the residues of the electrostatic loop (residues 121–142) of chain A (cyan), and the reduced form of apo wtSOD1 also with the application of a harmonic restraint (green).

**Fig 5:**
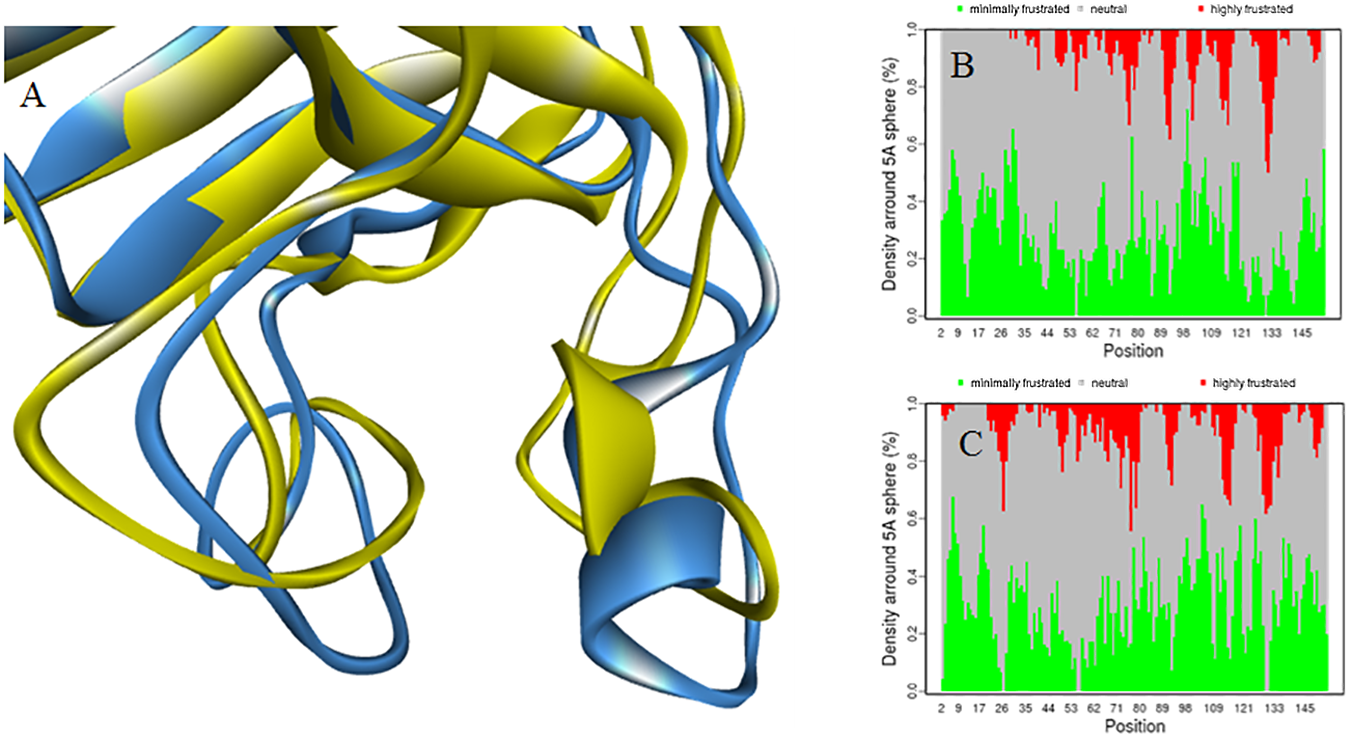
**A** Superimposition of the wtSOD1, yellow, with the structure for the reduced apo conformer of chain F that was generated by a 2ns MD production run with the electrostatic loop of chain A harmonically restrained, blue; Frustration contacts summarized as histograms of the distribution of the fraction of contacts in each of the highly, neutral and minimally frustration classes, found within a 5 Å sphere of each residue, for wtSOD1, **B,** and chain F of the reduced apo wtSOD1,**C.**

The root mean square fluctuations (RMSF) for 2 ns production runs of apo wtSOD1 (black), apo wtSOD1 with the application of a harmonic restraint to the residues of the electrostatic loop of chain A (cyan), and the reduced form of apo wtSOD1 also with the application of a harmonic restraint (green), is shown in Fig 4. The comparison clearly illustrates how constraining the electrostatic loop of one subunit dramatically increases the disorder in the electrostatic loop of the other subunit. Interestingly, constraining the electrostatic loop also enhances disorder within the zinc binding loop, but the correlated residues are different depending on whether the zinc binding loop is in the same, or the partner, subunit. Thus a complex such as that in Fig 1B, by enervating the electrostatic of the subunit at the dimer-dimer interface would be predicted to promote local unfolding of the zinc-binding loop within the subunit, and both the electrostatic and zinc-binding loops within of the partner subunit. For the apo form of SOD1 where the highly conserved Cys_57_-Cys_146_ disulfide bridge has been reduced residue fluctuations are greatly enhanced. The extent of the resulting conformational change facilitated by this reduction can be seen from the superimposition of the structure for wtSOD1, yellow in Fig 5A, with the structure for the reduced apo conformer of chain F generated by a 2ns MD production run with the electrostatic loop of chain A harmonically restrained, shown as blue in Fig 5A. Frustration contacts for the two conformers, summarized as histograms of the distribution of the fraction of contacts in each of the highly, neutral and minimally frustration classes, found within a 5 Å sphere of each residue, are shown in Fig 5B (wtSOD1) and Fig 5C (reduced apo wtSOD1). Calculations of local frustration performed on unbound monomers have shown that highly frustrated contacts both cluster and tend to be closer to binding residues than they are to other surface residues [28]. The frustration distributions in Fig 5B identify the electrostatic loop as a predicted point of contact for formation of any higher order oligomer.

**Fig 6:**
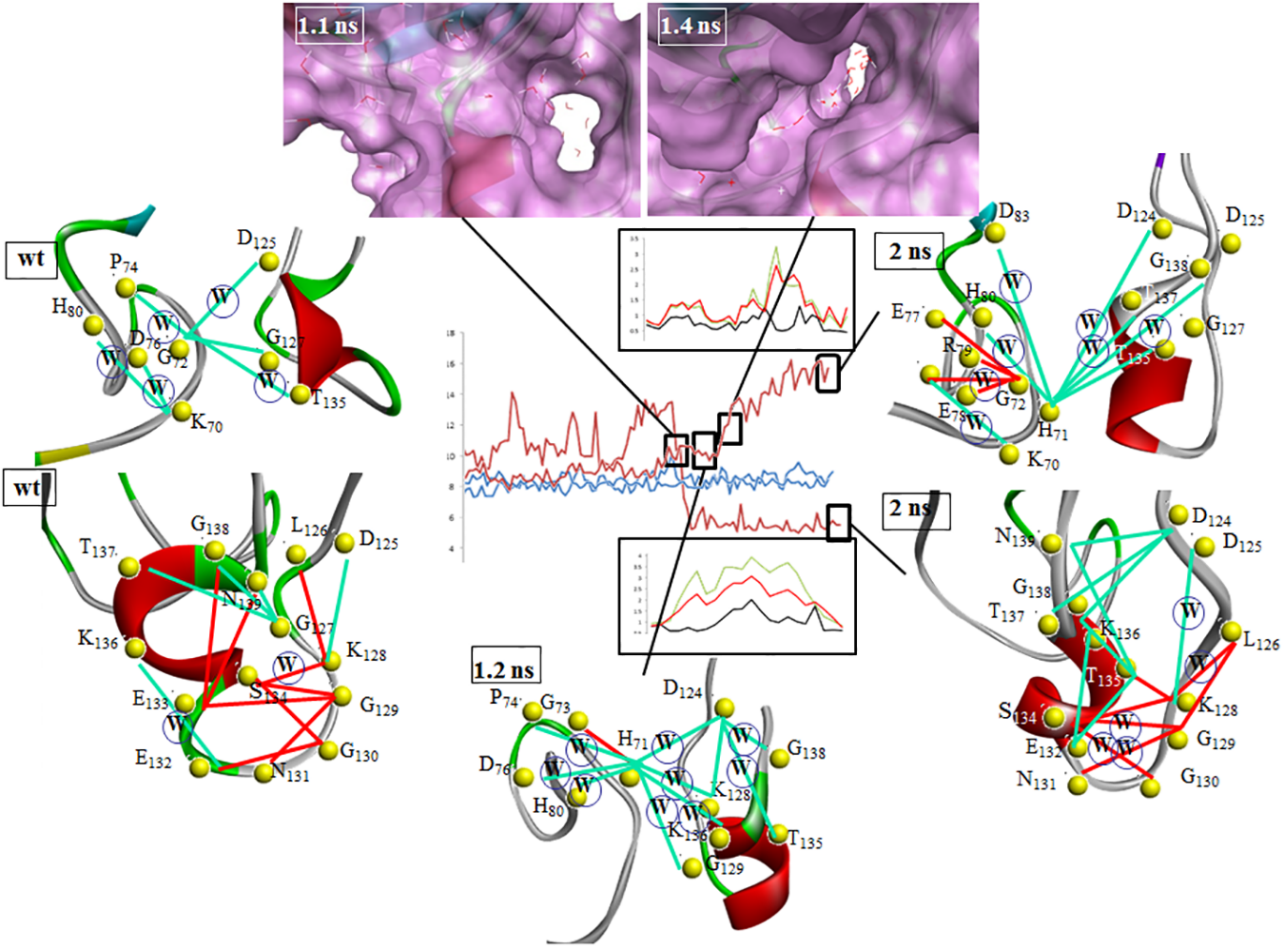
MD trajectory analysis for the Lys_128_-Ser_134_ and Asp_124_-His_71_ frustration contacts for apo wtSOD1 with no restraint (blue line) and chain F of the reduced apo wtSOD1 with the application of a harmonic restraint to the residues of the electrostatic loop of chain A (red line); Highly frustrated (red) and minimally frustrated (green) contacts localized to the electrostatic and zinc-binding loops for wtSOD1,the conformer at 1.2 ns for the apo reduced formed with restraint applied, and the conformer at 2 ns for the apo, reduced formed with restraint applied, where W signifies a water-mediated contact; Inset top compares the RMSF of the zinc-binding residues for the apo reduced form, with application of restraint, over the period following the conformational transitions (red) to the RMSF for those residues throughout the full 2ns trajectory (green), as well as to RMSF where no constraint was applied (black); Inset bottom does the same for the electrostatic loop; Solvent accessible surfaces for MD frames at inflection points leading to electrostatic loop and zinc-binding loop conformational changes.

Those highly frustrated (red) and minimally frustrated (green) contacts localized to the electrostatic and zinc-binding loops of the SOD1 that change in character during the course of the MD simulation are shown in Fig 6. Thermal fluctuations within the electrostatic loop that have previously been identified through NMR analysis are reflected in the nest of intra-loop highly frustrated contacts calculated for wtSOD1. These residual contacts facilitate a local motion that presumably serves to dissipate the energy associated with environmental perturbations. By contrast, the minimally frustrated contacts calculated for the zinc-binding loop characterize it as stable, immobilized in part by long-range interactions with select residues in the electrostatic loop. This distribution changes markedly for the reduced apo conformer generated by MD with constraint applied to the electrostatic loop residues of one subunit. In this structure the electrostatic loop is more compact, stabilized by minimally frustrated contacts between residues located towards the N- and C-termini, with frustration now localized at the base of the loop. By contrast, highly frustrated contacts now predominate within the zinc-binding loop, even as inter-loop stabilization is indicated by a series of minimally frustrated contacts at the His_71_ nexus. Trajectory analysis for selected water-mediated frustration contacts illustrate the key role played by solvation/desolvation in driving this conformational change. As evidenced by the blue line in Fig 6 the highly frustrated water-mediated contacts Lys_128_-Ser_134_ and Asp_124_-His_71_ oscillate around a mean distance over the course of the MD simulation of wtSOD1. Reducing the disulfide bridge and imposing constraints on the electrostatic loop of one subunit, however significantly amplifies the fluctuation around these contacts, as shown by the red line in the trajectory in Fig 6, ultimately leading to conformational changes in both loops. A transition of the Lys_128_-Ser_134_ contact from a highly frustrated water-mediated contact to just a highly frustrated contact around 1.2 ns results in a dramatic contraction of the inter-residue distance, resulting in a change to a more compact electrostatic loop. As shown by the frustration analysis of the structure from the MD snapshot frame such a change introduces a water-mediated Asp_124_-His_71_ contact, which though minimally frustrated is associated with greater mobility of the zinc-binding loop There follows at 1.4 ns a localized, but dramatic, conformational change of this loop, signified by a dramatic increase in the Asp_124_-His_71_ inter-residue distance. By comparing the RMSF over the period following these transitions (red in the inset boxes in Fig 6) to the RMSF throughout the full 2ns trajectory (green), as well as to RMSF where no constraint was applied (black), one can see that these conformational changes have different consequences for the mobility of the residues within these two secondary structures. For the residues in the zinc-binding loop (upper inset) nearly all of the fluctuation observed after imposition of the constraint is due to this conformational change. However the contraction at the base of the electrostatic loop has the opposite effect, dampening the residue fluctuation relative to that observed prior to the conformational switch.

**Fig 7:**
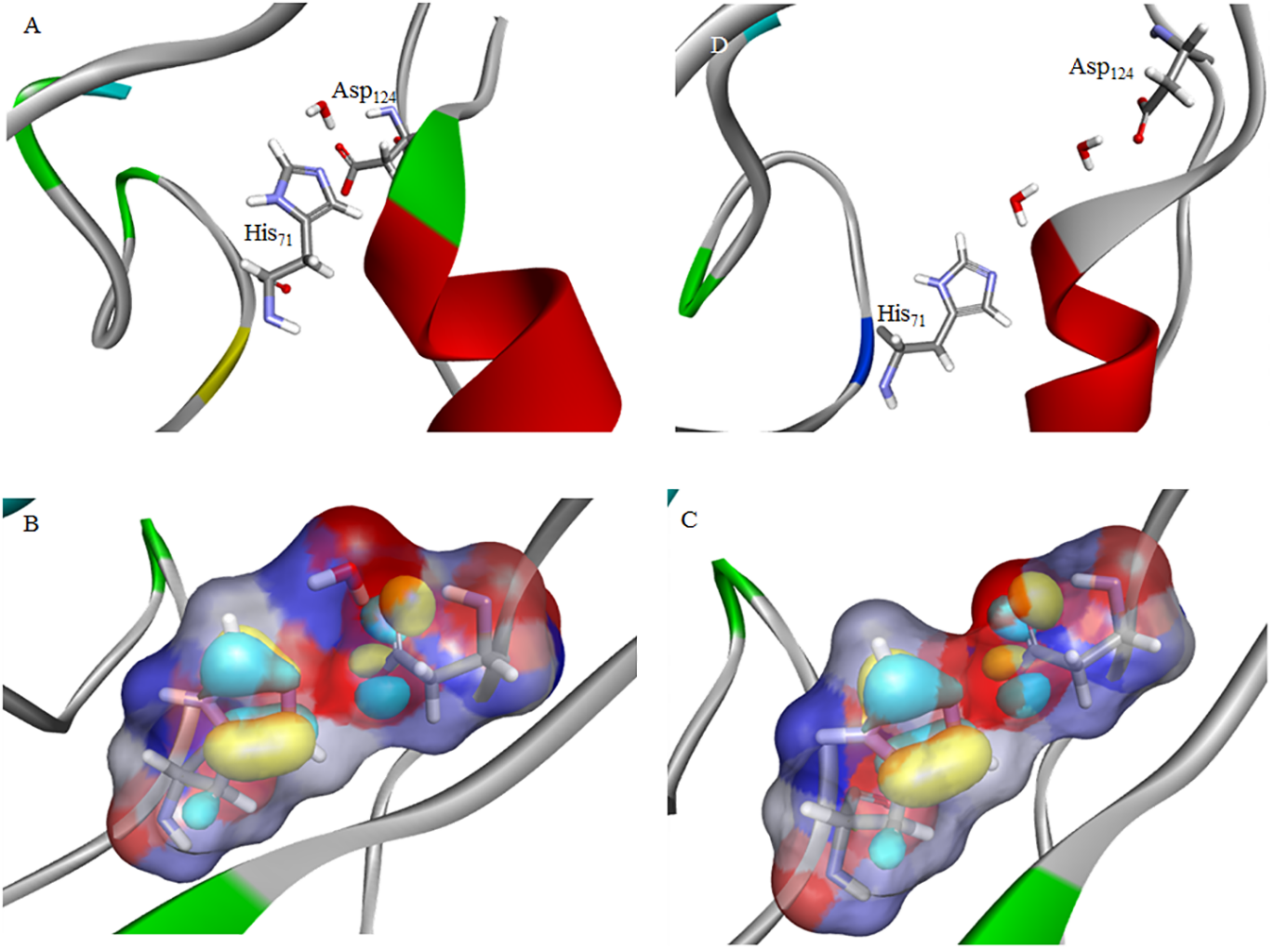
**A** MD snapshot frame at 1.2 ns for the reduced form of apo wtSOD1; **B** Electrostatic potential surface for the Asp_124_-His_71_ contact in the presence of the bound water molecule, with red signifying a negative potential and blue a positive potential. The highest occupied molecular orbitals (HOMO) of the Asp_124_ and His_71_ residues are shown in surface relief; **C** Electrostatic potential surface for the Asp_124_-His_71_ contact in the absence of the water molecule; **D** MD snapshot frame at 2 ns for the reduced form of apo wtSOD1.

Solvent accessible surfaces for MD frames at inflection points leading to electrostatic loop and zinc-binding loop conformational changes highlight the changing solvation patterns that accompany these conformational changes. Prior to 1.2 ns hydration of the electrostatic loop residues is facilitated by a solvent accessible intra-loop cavity, as shown in Fig 6. Conformational change leading to contraction at the base of the loop alters this surface, desolvating the electrostatic loop and solvating the zinc-binding loop, as seen in the cavity surface at 1.4 ns in Fig 6. This results most notably in the desolvation of an erstwhile water-mediated minimally frustrated contact that had been transiently formed between Asp_124_ and a newly proximate His_71_ residue. This water-mediated contact, predicted by frustration analysis, is confirmed by inspecting the MD snapshot frame at 1.2 ns, Fig 7A. A quantum mechanical (QM) electrostatic potential surface, Fig 7B, highlights the key role played by the bound water molecule in stabilizing this interaction. In the presence of the water molecule, the potential goes from negative (red) to positive (blue) to negative as one moves from the imidazole ring to the water to the carboxyl side-chain. Upon desolvation the highest occupied molecular orbitals (HOMO) of the Asp_124_ and His_71_ residues, shown in surface relief, ensure a destabilizing junction of two negative potentials, red in Fig 7C. The conformational change of the zinc-binding loop around 1.4 ns relieves this repulsive interaction, replacing it with a long-range water-mediated minimally frustrated contact between the same two residues. This prediction from frustration analysis is also borne out by an inspection of an MD snapshot frame at 2 ns, Fig 7D.

Primary coordinate analysis (PCA) of the MD trajectories of SOD1 mutants suggests that correlated motions of secondary elements within the SOD1 monomer, specifically the electrostatic loop and the zinc binding loop, allows for the packing strain induced by mutation to be propagated through the protein. However all the mutations studied disrupted a coupled motion between monomers, a correlated movement that is only found in wtSOD1 (The eigenmode from primary coordinate analysis (PCA) of the MD trajectory of wtSOD1 highlighting the correlated “breathing” motion of the electrostatic loops is available as s a movie (.mpg), S1 Movie as Supporting Information). This globally correlated movement represents a “breathing motion” of the electrostatic loops, a motion that is absent in all of the SOD1 mutants studied [14]. Thus if the tetramer in Fig 1B is formed between a partially demetallated, or reduced, wtSOD1 dimer and a pathogenic mutant, then the resulting enervation of the electrostatic loop at the dimer-dimer interface would disrupt this coupled motion. With the correlated motion between the subunits disrupted, the results summarized in Fig 6 would predict a local unfolding of the zinc binding and electrostatic loops within the partner subunit, thereby enhancing aggregation propensity. This result provides for a prion-like mechanism propagated through the PPIs at the interface of a mutant/wt SOD1 oligomer.

Prions are self-replicating misfolded proteins that serve as infectious agents through the formation of aggregates. As noted in reference [3] two possible prion-like mechanistic schemes have thus far been considered as possibly operating in ALS, one where misfolded SOD1 becomes more stable upon aggregation, and another where the more stable misfolded form becomes kinetically accessible through aggregation. While the results reported here do not allow for the delineation of a complete prion-like replication cycle they do hint at possible scenarios. The most compelling is one where the SOD1 mutant forms a non-obligate oligomer comprised of both mutant and wt SOD1 homodimers. Whereas the ensuing PPIs exert a stabilizing effect on the more flexible mutant, they initiate local conformation change in both the zinc-binding and electrostatic loops of the wt component. Upon release this locally misfolded, and more labile, wt SOD1 homodimer in turn seeds the formation of other non-obligate oligomers. The ensuing buildup of misfolded near-native conformers then is, as per our model, driven by harnessing the kinetic energy normally dissipated as a correlated motion of the electrostatic loops in the wt SOD1 homodimer. For this model to account for fibril formation more extensive dislocation of these loops is required. Quantum mechanical characterization of misfolded intermediary conformers points to the stabilizing effects of solvation as an additional element favoring prion-like aggregation. This in turn leads to a model that is partly kinetic and partly thermodynamic in character.

As noted above the localized unfolding represented by Fig 5A does not represent conformational change sufficient to facilitate the GOI characterized in the H46R filaments. However the mechanism involving transient oligomer formation does allow for the generation of locally unfolded near native SOD1 states, that if populated to a significant extent would be subject to macromolecular crowding effects. In developing an analytical function to model macromolecular crowding effects in protein folding the authors noted that populating non-native states so that they could contribute meaningfully to the crowding potential would require a lowering of their conformational free energy by “greater than about 3–4 kT” [29]. For the wtSOD1 structure intermediate between the electrostatic and zinc-binding loop conformational changes, shown as the MD snapshot frame at 1.2 ns in Fig 7A, we have calculated free energies of solvation of between -3.7 to -4.2 kcal mol^-1^. This stabilization of approximately 6–7 kT is more than sufficient to ensure a significant equilibrium conformer population. Thus solvation of these near-native conformers of unfolded SOD1 might allow them to contribute significantly to the Boltzmann-weighted average free energy of states, leading to the promotion and acceleration of pathological SOD1 oligomerization and aggregation by macromolecular crowding effects. It has been previously observed that the rate and extent of oligomer and fibril formation in crowded environments can differ by orders of magnitude from that observed *in vitro* [30], and more specifically crowding effects have also been shown to accelerate the fibrillization of α–synuclein [31,32].

Finally, dehydron analysis proves instructive in providing a structural understanding of how the β_6_-β_7_ loop region of SOD1 might initiate protein aggregation. The hydrogen bond within the _106_LSGDHC_111_ sequence, between the Cys_111_ donor amide and the Gly_108_ acceptor carbonyl, is underwrapped and thus vulnerable to solvent exposure. However in the helical amyloid filament of Zn-H46R it is shielded, or desolvated, by the β_6_-β_7_ loop of an adjacent dimer, shown in Fig 2A as spacefilled atoms. This hydrogen bond within the LSGDHC sequence is exposed in the dimer and can therefore be viewed as a “sticky” site within the β_6_-β_7_ loop, one that would benefit from interaction with a protomer, and can serve point of contact for protein aggregation as shown in Fig 2A.

## Supporting information

S1 Movie A movie (.mpg) of the eigenmode from primary coordinate analysis (PCA) of the MD trajectory of wtSOD1 highlighting the correlated “breathing” motion of the electrostatic loops. (MPG)
